# Mixture Effects of Polychlorinated Biphenyls (PCBs) and Three Organochlorine Pesticides on Cognitive Function in Mohawk Adults at Akwesasne

**DOI:** 10.3390/ijerph20021148

**Published:** 2023-01-09

**Authors:** Nozomi Sasaki, Laura E. Jones, Gayle S. Morse, David O. Carpenter, on behalf of the Akwesasne Task Force on the Environment

**Affiliations:** 1 Institute for Health and the Environment, University at Albany, Rensselaer, NY 12144, USA; 2 Department of Biostatistics and Epidemiology, School of Public Health, University at Albany, Rensselaer, NY 12144, USA; 3 Department of Psychology, School of Health Sciences, Russell Sage College, Troy, NY 12180, USA

**Keywords:** cognitive impairment, DSST, native americans, PCBs, HCB, DDE, mirex, mixture effects, g-computation

## Abstract

The Mohawks at Akwesasne have been highly exposed to polychlorinated biphenyls (PCBs), via releases from three aluminum foundries located near the reserve. They are also exposed to organochlorine pesticides, namely hexachlorobenzene (HCB), dichlorodiphenyldichloroethylene (DDE), and mirex. Previous studies have demonstrated reduced cognition in relation to total PCBs, but the effects of the mixtures of different PCB congener groups, HCB, DDE, and mirex on cognitive function have not been studied. Therefore, cognitive performance for executive function, scored via the digit symbol substitution test (DSST), in Mohawk adults aged 17–79 years (*n* = 301), was assessed in relation to serum concentrations of low-chlorinated PCBs, high-chlorinated PCBs, total PCBs, HCB, DDE, and mirex. We used mixture models employing the quantile-based g-computation method. The mixture effects of low-chlorinated PCBs, high-chlorinated PCBs, HCB, DDE, and mirex were significantly associated with 4.01 DSST scores decrements in the oldest age group, 47–79 years old. There were important contributions to mixture effects from low-chlorinated PCBs, high-chlorinated PCBs, and total PCBs, with smaller contributions of HCB and DDE. Our findings indicate that exposures to both low- and high-chlorinated PCBs increase the risk of cognitive decline in older adults, while DDE and HCB have less effect.

## 1. Introduction

Polychlorinated biphenyls (PCBs) were widely used in electric manufacture and in building materials in the past [1]. Dichlorodiphenyldichloroethylene (DDE) is the major degradant of dichlorodiphenyltrichloroethane (DDT), which was used for pest control in agricultural and residential areas. Mirex was used as a flame retardant and pesticide [2]. In the late 1970s, PCB, DDT, and mirex were banned from manufacturing in the U.S. [1,2] Hexachlorobenzene (HCB) is a fungicide used for seed treatment, and the use was ended in 1984 [3]. However, PCBs and organochlorine pesticides are persistent compounds, have lipophilic and bio-accumulative natures, and are still of concern for adverse health effects as both are found at measurable concentrations in everyone [4,5].

The Akwesasne Mohawks are an indigenous American people, living along the St. Lawrence River and located in upstate New York and the Canadian provinces of Quebec and Ontario. In the past, they practiced a traditional subsistence living, consuming local fish as a major protein source. The Akwesasne Reserve is immediately downriver from a U.S. federal Superfund site (the General Motors Central Foundry) and two NYS Superfund sites (Reynolds Metal Company and the Aluminum Company of America). Those local industrial sites discharged PCBs into the St. Lawrence River and its tributaries for more than two decades from 1959 to the 1980s [6,7]. Consequently, the Akwesasne Mohawks have been highly exposed to PCBs from ingestion and inhalation because PCBs contaminated local fish, wildlife, agricultural crops, soil, and ambient air. The New York State Health Department imposed a warning against eating locally caught fish in the 1980s, and the advisories are still in place [8]. 

In previous studies, PCB exposure in Mohawk adults has been associated with an increased risk of thyroid disease, reproductive abnormalities in both men and women, diabetes, hypertension, obesity, hyperlipidemia, and metabolic syndrome [9]. While earlier studies monitored total PCBs in blood, more recent studies investigated the effects of different PCB congener groups. This is important because high-chlorinated congeners are more persistent in both the environment and the human body, and their primary route of exposure is from the ingestion of animal fats, especially contaminated fish [10]. However, lower chlorinated congeners are more easily metabolized and, therefore, are less persistent and more volatile, leading to inhalation as a major route of exposure. We found that for some diseases, such as diabetes [11] and the abnormalities of menstruation and ovulation [12,13], the biological effects were primarily due to the low-chlorinated congeners. However, for other outcomes, such as hyperlipidemia and obesity, high-chlorinated congeners are more strongly associated, implicating ingestion as the major route of exposure [9]. 

PCB exposure also adversely affects cognitive function. Mohawk adolescents at Akwesasne were studied in an investigation of the adverse effects of PCB exposure on cognition, and it was found that the elevated concentrations of total PCBs were negatively associated with delayed recall, long-term retrieval, and comprehension knowledge scores [14]. Dioxin-like, non-dioxin-like, persistent, and low-persistent PCB congener groupings were all associated with reduced long-term memory [15]. Auditory processing was associated only with the persistent congener group, while the non-persistent congener group was associated with the scores of delayed recall, long-term retrieval, and comprehension knowledge [15]. Therefore, Mohawk adolescents who were highly exposed to PCBs showed significant detriments to cognitive skills. 

Haase et al. administered 18 different cognitive function tests to Mohawks between the ages of 18 and 79 and reported that total PCB concentrations were negatively associated with executive function, motor functioning, and memory [6]. There appeared to be a threshold in the total PCBs of around 2 ng/g. However, this study did not address the association between PCB congener groups, organochlorine pesticides, and their mixture effects on cognitive function. We used the data collected by Haase et al. to address these issues, using one of the tests, the digit symbol substitution test (DSST). Highly correlated environmental exposures, including PCB congener groups and organochlorine pesticides, may introduce multicollinearity into statistical models [16,17,18]. To date, studies reporting the effects of PCBs or organochlorine pesticides generally do not include co-exposures in their analyses or do not use models that handle random effects and interactions between PCB/organochlorine pesticides and co-exposures. The mixture analysis of PCBs and organochlorine pesticides may provide insight into the effects of these co-exposures. Therefore, the present study utilizes the data collected by Haase et al. to identify the mixture effects among exposures to congener groups comprising low-chlorinated PCBs (1–4 chlorinated PCBs), high-chlorinated PCBs (6–10 chlorinated PCBs), as well as total PCBs, HCB, DDE, and mirex, on cognitive function. PCB congener groups were categorized into low- and high-chlorinated PCBs based on their vapor pressure and evaporation rate, which is much greater for congeners with four or fewer chlorines than for those with six or more chlorines [10]. While Mohawk adults are not more highly exposed to organochlorine pesticides than the general U.S. population, another goal was to determine whether they influence the adverse cognitive effects of PCB exposure. We employed mixture modeling via the quantile-based g-computation method, which handles some multicollinearities via quantile transformation and allows flexible fitting, the directional heterogeneity of exposure components, and complex interactions [16,19]. The goals of the present study were two-fold: to investigate the associations between PCB/organochlorine pesticides and cognitive function in Mohawks and to identify whether low-chlorinated PCBs, for which inhalation is the major route of exposure, or high-chlorinated PCBs, for which ingestion is the major route of exposure, have greater deleterious effects on cognition.

## 2. Materials and Methods

### 2.1. Study Design and Population

We conducted a cross-sectional study that is an expanded analysis of the data collected by Haase et al. from 301 Akwesasne Mohawk adults for whom serum concentrations of PCBs, HCB, DDE, and mirex were measured and who were evaluated for cognitive function with DSST [6]. The age range of these participants was 17–79 years old.

### 2.2. DSST Module

In the clinical setting, DSST is used for evaluating cognitive impairment in relation to processing speed, motor ability, attention, visuoperceptual functions, working memory, and executive function [20]. The Wechsler Adult Intelligence Scale III DSST was administered by trained Mohawk staff at Akwesasne [21]. The test consists of a series of nine numbers paired with symbols. Participants are given only two minutes to copy the appropriate symbols into blank spaces with numbers on a 133-box grid. The score is the total number of boxes with the correct markings (range: 0 to 133).

### 2.3. Assessment of PCB and Organochlorine Pesticides Concentrations

Blood samples were collected by trained Mohawk staff at a project laboratory located within the Akwesasne Reserve or at the study participants’ homes. The participants were asked not to eat or drink anything after 10 PM on a preceding evening. Approximately 20 mL of blood was collected from each subject and sent to the analytical laboratory at the Institute for Health and the Environment at the University at Albany. An ultra-trace analytical method using dual-column gas chromatography with electron-capture detection was employed to determine 91 analytical peaks (representing a total of 101 PCB congeners) plus HCB, DDE, and mirex, in 5 g serum specimens. An analysis was performed concurrently on DB-5 and Apiezon-L capillary columns configured in parallel. Details on sample processing, the analytical method for the quantitation of individual congeners, and laboratory quality assurance/quality control procedures have been previously published [22]. Method detection limits (MDLs) for PCBs were in the range of 0.01–0.04 ng/g serum and MDLs for HCB, DDE, and mirex were 0.02 ng/g serum [22,23]. 

We created PCB congener groups based on the numbers of chlorines and calculate their summed concentrations. Low-chlorinated PCBs that can be inhaled are those PCBs with one to four chlorines, including PCB1, 2, 3, 4, 6, 7, 8, 9, 10, 13, 15, 16, 17, 18, 19, 22, 24, 25, 26, 27, 28, 29, 31, 32, 33, 40, 42, 44, 45, 46, 47, 49, 51, 52, 53, 56, 59, 63, 64, 66, 67, 70, 71, 74, and 77. People are exposed to PCBs with more than four chlorines primarily, but not exclusively, through ingestion. We considered high-chlorinated PCBs to be those congeners with six to ten chlorines, including PCB 128, 129, 130, 132, 134, 136, 137, 138, 141, 144, 146, 147, 149, 151, 153, 156, 158, 163, 164, 170, 171, 172, 174, 176, 177, 179, 180, 183, 185, 187, 190, 194, 195, 196, 199, 200, 201, 203, and 206. Total PCBs summed all the detected PCBs: PCB 1, 2, 3, 4, 6, 7, 8, 9, 10, 13, 15, 16, 17, 18, 19, 22, 24, 25, 26, 27, 28, 29, 31, 32, 33, 40, 42, 44, 45, 46, 47, 49, 51, 52, 53, 56, 59, 63, 64, 66, 67, 70, 71, 74, 77, 83, 84, 87, 90, 91, 92, 95, 97, 99, 101, 105, 109, 110, 114, 118, 123, 128, 129, 130, 132, 134, 136, 137, 138, 141, 144, 146, 147, 149, 151, 153, 156, 158, 163, 164, 170, 171, 172, 174, 176, 177, 179, 180, 183, 185, 187, 190, 194, 195, 196, 199, 200, 201, 203, and 206.

### 2.4. Statistical Analyses: Analyses of Exposures to PCBs or Organochlorine Pesticides on Cognitive Function

The correlations between DSST scores and low-chlorinated, high-chlorinated, and total PCBs, HCB, DDE, mirex, and participant fish consumption were assessed via Spearman’s rank correlations. Spearman’s rank correlations simply measure the strength and direction of the monotonic relationship between two variables. It cannot be applied to measure the associations between two variables due to categorical variables and possible outliers. Then, the distributions of the DSST relative to the three PCB groups, HCB, DDE, and mirex, were assessed. In the analyses, contaminate concentrations were log-transformed. We then performed linear regression to identify the associations between exposures to a single PCB congener group or organochlorine pesticide and DSST scores. Finally, a mixture analysis was performed. Potential confounders were chosen by using the appropriate directed acyclic graphs (DAGs) for each model family, sex at birth (male, female), education level (high school graduate, at least some college), and age (18–27, 28–35, 36–46, and 47–79 years old). Quartiles were selected for age categorization in order to perform even sampling in a relatively small and non-uniformly distributed age cohort. 

### 2.5. Statistical Analyses: Mixture Analysis of PCBs and Organochlorine Pesticides on Cognitive Function

We assessed the joint effects of the mixtures of PCB congener groups, HCB, DDE, and mirex, using the quantile-based g-computation method [19]. This method is appropriate for mixture models, where the directional heterogeneity of correlated environmental exposures on outcomes is identified. Additionally, estimated weights indicate the contributions of each chemical component to the mixture’s effects on outcomes. However, this method is limited for application to dose–response curves and marginal structural models and may not capture the dose–response function [19]. The method workflow proceeds as follows: Exposure data were standardized and transformed into quantiles. We fit a generalized linear model that estimates the individual effects of each mixture element on the outcome and then makes predictions at each quantile level of the exposures. A marginal model was fit for these predictions, with the result being an estimate of the outcome associated with a one-quartile increase in the exposure mixture levels. Multivariate regression with a Gaussian link function was used to examine the associations between DSST scores and a mixture of low-chlorinated PCBs, high-chlorinated PCBs, HCB, DDE, and mirex (hereafter “Model 1”), or a mixture of total PCBs, HCB, DDE, and mirex (“Model 2”), adjusted for potential demographic and behavioral confounders. Note that we created two model families to assess the mixture effect of the summed PCBs versus the components’ mixture effects of specific congener groupings. All significant potential confounders were determined from demographic analysis and confirmed via DAGs. Statistical analyses were performed using R version 4.1.0 (R Core Team, Vienna, Austria), and the mixture analysis was performed in R, using the package qgcomp [19].

## 3. Results

### 3.1. Overview of the Study Population

**Figure 1:**
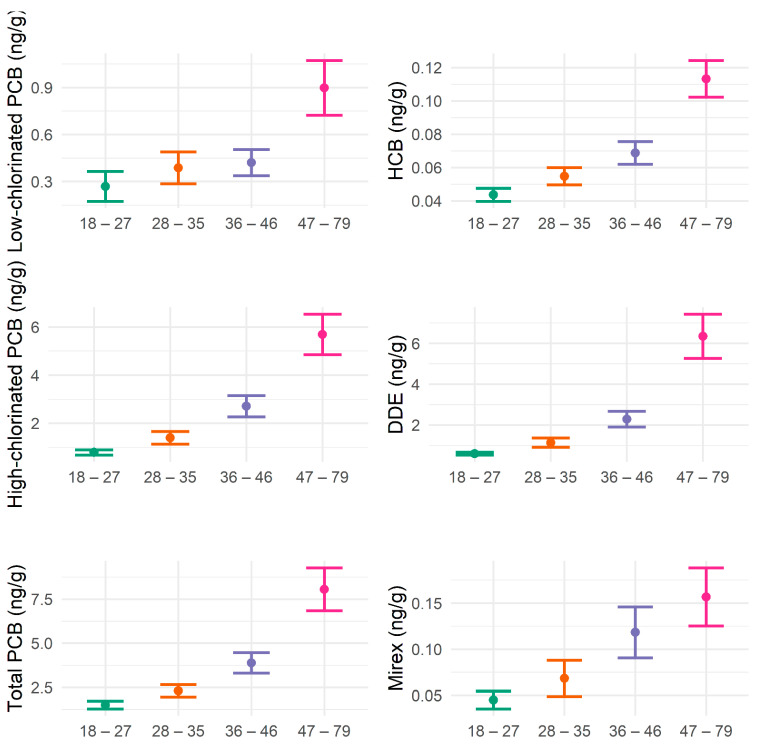
Means with 95% confidence intervals of serum concentrations of low-chlorinated PCBs, high-chlorinated PCBs, total PCBs, HCB, DDE, and mirex by age groups (years old) in Mohawk adults. Green color indicates the age group of 18 to 27 years old, orange is the 28 to 35 years old group, purple is the 36 to 46 years old group, and pink is the 47 to 79 years old group.

**Figure 2:**
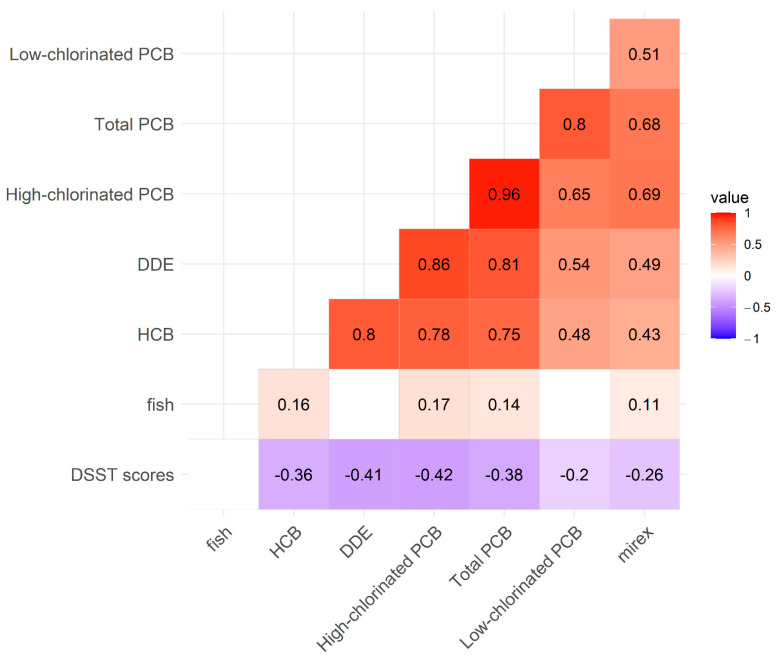
Spearman’s rank correlations between PCBs congener groups, HCB, DDE, mirex, fish consumption groups, and DSST in Mohawks. Red color indicates significant positive correlations between two variables, and dark red means high correlations, close to 1.0. On the other hand, blue color indicates negative correlations, and those in dark blue are highly negative correlations, close to −1. Blank cells are not significant correlations and correlation coefficient of rho is the number in each cell. All toxicants had weak or moderate negative correlations with DSST scores.

A total of 301 Mohawk adults completed PCB biomonitoring and the DSST module. The distributions of cognitive scores varied among subgroups by age, sex, and education level (Table 1). The mean DSST scores were lower in older, male, and less educated groups, while smoking and local fish consumption were not significantly different in subgroups (Table 1). The mean concentrations of low-chlorinated PCBs, high-chlorinated PCBs, total PCBs, HCB, DDE, and mirex in serum increased with age (Table 2, Figure 1). Serum concentrations of low-chlorinated PCBs, high-chlorinated PCBs, total PCBs, DDE, HCB, and mirex, were highly positively correlated with each other (ρ ≥ 0.60), while fish consumption showed a weak positive correlation with some pollutants (Figure 2).

### 3.2. Associations between Single PCB Congener Groups or Organochlorine Pesticide Exposures and Cognition

Single pollutant regressions showed significant and negative associations only for HCB and DDE, but negative associations for HCB were barely significant (Table 3). However, all the pollutants except for mirex showed negative associations with DSST scores, while mirex was weakly positive. The adjusted model for fish consumption, BMI, and smoking had little effect on the estimates.

### 3.3. Mixture Effects of PCBs and Organochlorine Pesticides on Cognition

**Figure 3:**
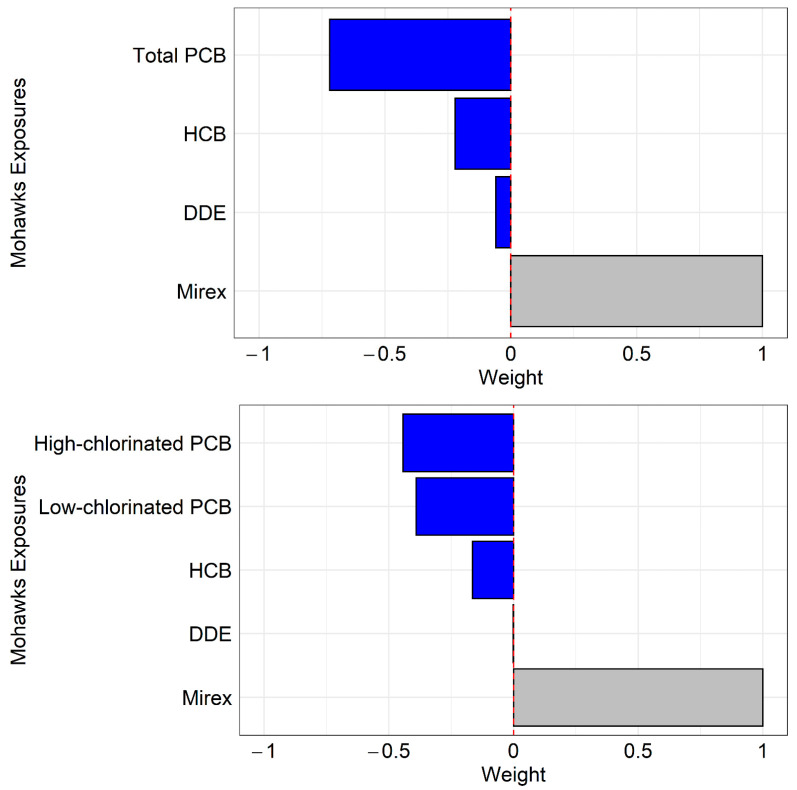
Component weights from the quantile-based g-computation method in the oldest age group. The top figure is from the mixture model with total PCBs, HCB, DDE, and mirex. The bottom figure is from the mixture model with low-chlorinated PCBs, high-chlorinated PCBs, HCB, DDE, and mirex. Models were adjusted for sex and education levels. Significant component weights are shown in blue, and nonsignificant components are shown in gray.

The mixture effects from the quantile g-computation method showed significant negative associations on DSST in the oldest age group, which decreased by 4.01 points for every unit increase in low-chlorinated PCBs, high-chlorinated PCBs, HCB, DDE, and mirex (Model 1) and declined by 3.72 points for total PCBs, HCB, DDE, and mirex (Model 2) (Table 4). In Model 1, component weights were heavily negative with high-chlorinated PCBs and low-chlorinated PCBs, while HCB and DDE were less weighted (Figure 3). Similarly, in Model 2, total PCBs were heavily weighted, but HCB and DDE were weakly weighted (Figure 3). In both Models 1 and 2, mirex showed positive weights, which did not significantly contribute to the mixture effects. There were no significant associations in younger age groups (Table 4).

## 4. Discussion

### 4.1. Study Population

Mohawk adults in the age group 47–79 years old have had prolonged exposure over time to PCBs, HCB, DDE, and mirex, and this exposure correlates with decreased cognitive function. When the oldest age group was compared with the youngest age group aged 18–27 years, they showed a 4.46-fold higher concentration of low-chlorinated PCBs, 7.01-fold for high-chlorinated PCBs, 5.42-fold for total PCBs, 2.75-fold for HCB, 8.59-fold for DDE, and 5.50-fold for mirex, respectively (Table 2). This is for two reasons. Older people were around during the period in which PCBs and organochlorine pesticides were being manufactured and widely used. Furthermore, the rate at which PCBs are metabolized in humans is slower than the rate at which they are taken in, so it is well known that concentrations increase with age [11]. Low-chlorinated PCB congeners are not as persistent as those with more chlorinated PCBs, as reflected by the 7.01-fold greater concentrations of high-chlorinated PCBs seen in older compared with younger Mohawks. It is possible that we underestimated the effects of the low-chlorinated PCBs. If inhalation exposure is essentially continuous, these lower chlorinated congeners may have effects not reflected by serum concentrations.

In the past, most studies of the health effects of PCBs monitored only total PCB concentrations. However, it is now clear that health effects are not necessarily associated only with persistence. Some of the adverse health effects are due to metabolites [24,25]. The PCBs stored in body fat are bioaccessible and reflect accumulation over a period of years. Organochlorine compounds are primarily metabolized by cytochrome P450 enzymes in the liver and other tissues, but the ability of these systems to destroy the molecules is reduced depending upon how many chlorines are found on the molecule, and where the chlorines are located. For PCBs, this results in a much more rapid metabolism of those congeners with fewer chlorines. Those congeners with only one or two chlorines are rarely found in serum samples at concentrations above the limit of detection. Some of those congeners with three or four congeners, especially those with a 4,4′ chlorine substitution, are volatile, and inhalation is a significant route of exposure, but because of the placement of the chlorines around the molecule, they are somewhat persistent. This has been discussed in detail in a publication by Casey et al. [26]. At least for some diseases, the lower-chlorinated PCB congeners that are more rapidly metabolized may have a greater impact on human health than those more persistent PCB congeners that are stashed away in body fat. This has been clearly shown for type 2 diabetes, menstrual irregularities, and ovulation, where it is the low-chlorinated PCB congeners that pose the greater risk [27]. This is the case even though serum analysis is clearly not a perfect measure of total exposure due to the more rapid metabolism of low-chlorinated PCBs. 

### 4.2. Exposure Route for PCBs, HCB, DDE, and Mirex

Among the Mohawks, low-chlorinated PCBs, high-chlorinated PCBs, and total PCBs were highly positively correlated (Spearman’s ρ range = 0.65, 0.96), and total PCBs and high-chlorinated PCBs were also highly correlated with DDE, HCB, and mirex (Spearman’s ρ range = 0.68, 0.86) (Figure 2). These results suggest that PCBs, DDE, HCB, and mirex migrate together and accumulate in the body. Fish consumption is a likely exposure route given Mohawk culture and diet [28], but the correlations between fish consumption and PCBs and organochlorine pesticides were weak (Spearman’s ρ range = 0.11, 0.16). In our previous research with NHANES data, fish consumption was associated with better DSST scores in older adults [29]. However, in the Mohawk population, fish consumption was not positively associated with DSST scores. This suggests that consuming locally caught fish, which contains high concentrations of PCBs, does not provide beneficial effects on cognitive function. Since the Mohawks have been highly contaminated with PCBs, exposure routes need further research, which should include ambient air, locally grown crops, locally caught wildlife, and drinking water. It is important to note that while significant concentrations of organochlorine pesticides, especially DDE and HCB, are present in the Mohawks, they are not present at concentrations that are different from that of the general U.S. population [23]. 

### 4.3. Evidence That PCB Exposure Results in Reduction in Cognitive Function

There is strong evidence that early life exposure to PCBs results in reduced IQ and neurobehavioral abnormalities [30]. Our colleagues have studied cognitive function in the Mohawks in relation to increasing PCB concentrations in adolescents, ages 10 to 16. In these cross-sectional studies of Mohawk adolescents, PCB exposures resulted in a significant decline in long-term memory, delayed recall, comprehension, and knowledge [14,15]. 

There has been less research on the cognitive effects of PCBs in adults, but epidemiologic studies have demonstrated the neurotoxic effects of PCB exposures on executive function and delayed memory in adults as well. Schantz et al. studied a population of adults that ate contaminated fish from Lake Michigan and were too old to have been exposed in childhood [31]. PCB exposures from locally caught fish consumption were associated with impairment in delayed memory and verbal learning memory in the cohort aged 49 to 89 years, but DDE was not significantly associated with memory impairment. In a Taiwanese cohort study, PCB-exposed women aged 60 years and older showed reduced attention, visual memory, and learning ability that was not observed in exposed men [32]. In Mohawk adults, total PCB concentrations were significantly negatively related to executive function, motor functioning, and memory, with a threshold dosage of about 2 ppb in older adults aged 37–79 years [6]. 

Cellular and animal studies have demonstrated several mechanisms responsible for neurotoxicity [33]. Lower chlorinated PCB congeners, especially those with multiple ortho-substituted chlorines, interfere with the synthesis of the important neurotransmitter, dopamine, and efficiently inhibit neurotransmitter uptakes [34,35]. These low-chlorinated PCB congeners also have cytotoxic effects, causing the generation of reactive oxygen species, an increase in intracellular calcium, and an increase in membrane fluidity [36]. These actions were not seen with those PCBs with more chlorines, nor with dioxin-like congeners. In addition, Aroclor 1248, which was the commercial PCB mixture used at Akwesasne, showed more cytotoxic actions on isolated neurons than another more highly chlorinated PCB mixture, Aroclor 1260 [37]. 

Long-term potentiation (LTP) is an electrophysiological response that is known to be an initial event in learning and memory formation. LTP in rodent brain slices is blocked by low concentrations of PCBs, including lower-chlorinated, high-chlorinated, and dioxin-like PCB congeners [38,39,40]. PCB-exposed adult rats accumulated more high-chlorinated PCBs than low-chlorinated PCBs in the brain [41], reflecting the more rapid metabolism of low-chlorinated congeners. These results suggest that both low- and high-chlorinated PCB congeners contribute to decrements in cognitive function and are consistent with our results of component weights in the mixture analysis.

### 4.4. Do Organochlorine Pesticides Contribute to Cognitive Decline?

There have been a number of reports indicating a decrement in cognitive function associated with exposure to chlorinated pesticides. Costa Rican DDT-exposed older adults were moderately associated with lower cognitive scores (*p* = 0.09), but no associations were found between DDE exposures and cognitive scores [42]. In a cross-sectional study comprising older NHANES adults from 1999 to 2002, the highest quartile groups of DDT, DDE, β-hexachlorocyclohexane, and HCH showed two to three times higher risk of lower cognitive scores [43]. These studies, however, did not measure PCBs that may have been responsible for the observed effects. A recent review in the Environmental Influences on Child Health Outcomes study concluded that findings pointing to the correlations between neurobehavioral outcomes and exposure to organochlorine pesticides are not consistent across age, groups, outcomes, or specific organochlorine pesticides [18]. Therefore, the associations between organochlorine pesticides and cognitive decline need further research to fully understand their extent and mechanisms.

### 4.5. What Is the Relative Contribution of Low- vs. High-Chlorinated PCBs to the Decrement in Cognitive Function?

Our findings from mixture analyses showed significant negative associations between the mixture of PCBs and organochlorine pesticides in older Mohawks. Importantly, in older Mohawks, low-chlorinated PCBs, high-chlorinated PCBs, and total PCBs were the most influential factors in cognitive decline, while HCB and DDE were moderate contributors (Figure 3). Our results cannot exclude the possibility that organochlorine pesticides, especially DDE and HCB, had some contribution to a reduction in cognitive function in the older group, but were in general consistent with the body of evidence that PCB exposure is much more important. As both groups of chemicals migrate together in food, especially in all animal fats, there is no human population exposed to only one group [18]. The use of mixture analysis helps to identify relative importance. Our results from component weights suggest that low-chlorinated PCB exposures via inhalation may be at least as important for cognitive decline as high-chlorinated PCBs because they showed similar component weights.

Several studies suggested the mixture of PCBs and organochlorine pesticides may increase neurotoxicity and the risk of cognitive decline. In adults aged 65 years or more, a Canadian national cohort study showed significant associations between PCBs, DDT, and DDE exposures and lower cognitive scores, in particular, with dementia [44]. Bowers et al. reported that a mixture of PCBs and 11 organochlorine pesticides, including HCB and DDE, suppressed neurodevelopment in rats, and the mixture was found to be more neurotoxic than exposure to PCBs alone [45]. Prenatal and postnatal exposures to a mixture of PCBs, organochlorine pesticides, including HCB and DDE, and methylmercury changed gene expression of the rat cerebellum in Purkinje cells, oligodendrocytes, and astrocytes, and these effects varied by sex [46]. However, because all of these organochlorine chemicals show high correlations, simply finding an association does not eliminate the possibility that the effect is due to a different chemical. The mixture effects of PCBs and organochlorine pesticides need further study due to possible additive and synergistic effects leading to neurotoxicity.

### 4.6. Study Strengths and Limitations

As data were obtained at one point in time, we could not assess temporal changes in cognitive function or pollutant exposures. We used good assessment methods to yield accurate data with confirmed QA/QC, but the total exposure to low-chlorinated PCBs was almost certainly underestimated due to their lower persistence in the human body. This study is an assessment of the associations between exposures and cognition in Mohawk participants, not a calculated prevalence ratio of cognitive impairment. We conducted analyses with a complete case dataset, omitting those individuals with missing units. Note that data can be assumed to be missing at random; if missingness is less than 10% of the sample, and if the remaining sample size is large enough for analysis, complete case analyses produce unbiased estimates [47].

## 5. Conclusions

We investigated exposures to low-chlorinated PCBs, for which inhalation is the most important route of exposure, high-chlorinated PCBs, for which ingestion is the most important route of exposure, total PCBs, and three organochlorine pesticides, and their mixture effects on cognitive scores in Mohawk adults. The mixture effects of these toxicants were significantly associated with DSST scores decrements in the oldest age group, 47–79 years old, who had two to nine times higher concentrations of PCBs and organochlorine pesticides in their blood than those seen in younger individuals. Given their chronic exposure to PCBs, important contributors to mixture effects were primarily low-chlorinated PCBs, high-chlorinated PCBs, and total PCBs, with a small impact from HCB and DDE. Older Mohawk adults that had elevated body burdens of PCBs are at significantly increased risk of cognitive decline. To our knowledge, this is the first study addressing the mixture effects of PCBs and organochlorine pesticides on cognitive decline in a heavily exposed population of Mohawks adults. 

## Figures and Tables

**Table 1:** Mean scores of DSST by demographic in Mohawk adults.

Variables	*n* (301)	DSST Scores (95% CI)	*p*-Value ^2^
Age categories, years old			
18–27	71	63.41 (60.68, 66.08)	0.97
28–35	71	64.41 (61.27, 67.54)	ref ^1^
36–46	81	55.58 (52.75, 58.41)	<0.01 ^3^
47–79	78	47.42 (44.06, 50.79)	<0.01 ^3^
Sex			
Male	102	50.52 (47.86, 53.18)	ref ^1^
Female	199	60.91 (58.93, 62.89)	<0.01 ^2^
Education			
≤High school graduate	134	51.03 (48.59, 53.47)	ref ^1^
≥Some college	167	62.49 (60.48, 64.50)	<0.01 ^2^
Smoking per day			
Nonsmoker	101	58.57 (55.48, 61.66)	ref
<1 pack	193	57.17 (55.15, 59.20)	0.37 ^2^
1–2 pack	7	46.29 (33.69, 58.88)	0.09 ^2^
Local fish consumption			
Yes	130	55.90 (53.31, 58.49)	0.13
No	171	58.52 (56.31, 60.73)	ref

^1^ ref, reference. ^2^ *p* value was assessed with *t* test. ^3^ *p* value was assessed with ANOVA and Tukey test.

**Table 2:** Distribution of low-chlorinated PCBs, high-chlorinated PCBs, total PCBs, HCB, DDE, and mirex in Mohawk nation participants (*n* = 301) by age group.

	Minimum	5th Percentile	25th Percentile	Median	Mean	75th Percentile	95th Percentile	99th Percentile	Maximum
Age group 18–27 (*n* = 71)									
Low-chlorinated PCBs, ng/g	0.02	0.03	0.08	0.13	0.27	0.26	1.11	1.83	1.86
High-chlorinated PCBs, ng/g	0.19	0.27	0.46	0.70	0.79	0.95	1.65	2.31	2.32
Total PCBs, ng/g	0.35	0.43	0.92	1.22	1.50	1.84	3.43	4.26	5.04
HCB, ng/g	0.02	0.02	0.03	0.04	0.04	0.05	0.07	0.08	0.09
DDE, ng/g	0.11	0.27	0.39	0.56	0.60	0.76	1.15	1.35	1.43
Mirex, ng/g	0.02	0.02	0.02	0.02	0.05	0.07	0.11	0.19	0.20
DSST	36.00	45.50	57.50	63.00	63.38	70.50	81.50	85.60	87.00
Age group 28–35 (*n* = 71)									
Low-chlorinated PCBs, ng/g	0.02	0.05	0.11	0.22	0.39	0.43	1.18	1.72	2.51
High-chlorinated PCBs, ng/g	0.31	0.49	0.77	1.14	1.39	1.59	3.75	5.68	6.41
Total PCBs, ng/g	0.49	0.85	1.26	2.03	2.30	2.57	5.23	7.49	8.06
HCB, ng/g	0.03	0.03	0.04	0.05	0.05	0.06	0.10	0.14	0.16
DDE, ng/g	0.32	0.49	0.65	0.87	1.13	1.21	2.67	4.66	6.74
Mirex, ng/g	0.02	0.02	0.02	0.04	0.07	0.07	0.19	0.44	0.45
DSST	26.00	47.00	57.00	63.00	64.41	73.00	87.00	89.20	92.00
Age group 36–46 (*n* = 81)									
Low-chlorinated PCBs, ng/g	0.02	0.05	0.16	0.30	0.42	0.60	1.12	1.39	2.16
High-chlorinated PCBs, ng/g	0.55	0.74	1.40	1.87	2.71	3.59	6.39	8.96	10.45
Total PCBs, ng/g	0.76	1.22	1.90	3.11	3.88	4.89	9.19	11.55	12.33
HCB, ng/g	0.02	0.03	0.05	0.06	0.07	0.08	0.13	0.17	0.19
DDE, ng/g	0.08	0.74	1.14	1.62	2.29	2.88	5.37	8.28	10.76
Mirex, ng/g	0.02	0.02	0.03	0.07	0.12	0.17	0.36	0.53	0.55
DSST	29.0	37.0	45.0	55.0	55.58	66.0	73.0	81.0	89.0
Age group 47–79 (*n* = 78)									
Low-chlorinated PCBs, ng/g	0.12	0.17	0.40	0.58	0.90	1.17	2.81	3.26	3.59
High-chlorinated PCBs, ng/g	0.98	2.36	3.27	4.91	5.69	6.71	12.06	20.61	20.94
Total PCBs, ng/g	1.67	2.96	4.73	6.62	8.06	9.37	18.76	28.68	28.77
HCB, ng/g	0.03	0.05	0.08	0.11	0.11	0.13	0.22	0.24	0.26
DDE, ng/g	0.64	1.40	2.80	4.81	6.35	8.37	16.05	21.76	22.51
Mirex, ng/g	0.02	0.03	0.07	0.11	0.16	0.19	0.45	0.65	0.68
DSST	9.00	23.25	39.25	45.50	47.42	59.00	68.00	83.38	88.00

**Table 3:** Results of linear regression for DSST regressed on log-transformed low-chlorinated PCBs, high-chlorinated PCBs, total PCBs, HCB, DDE, and mirex, adjusted for confounders (single pollutant models).

	Full Model Adjusted for Fish Consumption ^1^ (*n* = 291)		Full Model ^2^ (*n* = 301)	
	β (95% CI)	*p*-Value	β (95% CI)	*p*-Value
Low-chlorinated PCBs, ng/g ^3^	−0.39 (−1.94, 1.16)	0.62	−0.33 (−1.86, 1.20)	0.67
High-chlorinated PCBs, ng/g ^3^	−2.03 (−4.26, 0.21)	0.13	−2.00 (−4.19, 0.20)	0.07
Total PCBs, ng/g ^3^	−1.54 (−3.55, 0.48)	0.11	−1.52 (−3.50, 0.47)	0.13
HCB, ng/g ^3^	−1.90 (−3.71, −0.08)	0.04	−1.78 (−3.55, −0.01)	0.05
DDE, ng/g ^3^	−2.46 (−4.64, −0.28)	0.03	−2.35 (−4.50, −0.20)	0.03
Mirex, ng/g ^3^	0.13 (−1.48, 1.75)	0.87	0.10 (−1.48, 1.69)	0.90

^1^ The models were adjusted for age category, sex, BMI, education levels, smoking, and fish consumption group. ^2^ The models were adjusted for age category, sex, and education levels because BMI, smoking, and fish consumption were not significant confounders. This model had a slightly larger r^2^. ^3^ Serum concentrations.

**Table 4:** Mohawk adults from the age-stratified quantile g-computation method of Model 1, including congener groups of high-chlorinated PCBs, low-chlorinated PCBs, HCB, DDE, and mirex, and Model 2, including total PCBs, HCB, DDE, and mirex. Models 1 and 2 were adjusted for sex and education levels. All age models were adjusted for age category, sex, and education levels.

	All Age Model (*n* = 301)		Model Age 18–27 (*n* = 71)		Model Age 28–35 (*n* = 71)		Model Age 36–46 (*n* = 81)		Model Age 47–79 (*n* = 78)	
	β (95% CI)	*p*-Value	β (95% CI)	*p*-Value	β (95% CI)	*p*-Value	β (95% CI)	*p*-Value	β (95% CI)	*p*-Value
Model 1 ^1^										
Mixture	−1.06 (−3.61, 1.48)	0.41	1.45 (−6.85, 9.74)	0.73	1.33 (−3.01, 5.67)	0.55	0.18 (−2.71, 3.07)	0.90	−4.01 (−7.55, −0.47)	0.03
Model 2 ^2^										
Mixture	−1.03 (−3.55, 1.49)	0.42	0.09 (−7.61, 7.79)	0.98	0.18 (−3.92, 4.28)	0.93	0.16 (−2.66, 2.98)	0.91	−3.72 (−7.19, −0.25)	0.04

^1^ Model 1: low-chlorinated PCBs, high-chlorinated PCBs, HCB, DDE, and mirex mixture. ^2^ Model 2: total PCB, HCB, DDE, and mirex mixture.

## Data Availability

Not applicable.
